# A Bayesian approach for accurate de novo transcriptome assembly

**DOI:** 10.1038/s41598-021-97015-x

**Published:** 2021-09-03

**Authors:** Xu Shi, Xiao Wang, Andrew F. Neuwald, Leena Halakivi-Clarke, Robert Clarke, Jianhua Xuan

**Affiliations:** 1 Bradley Department of Electrical and Computer Engineering, Virginia Polytechnic Institute and State University, 900 North Glebe Road, Arlington, VA 22203 USA; 2 Institute for Genome Sciences and Department Biochemistry and Molecular Biology, University of Maryland School of Medicine, 670 W. Baltimore Street, Baltimore, MD 21201 USA; 3 Hormel Institute, University of Minnesota, 16th Street N, Austin, MN 55912 USA

**Keywords:** Computational biology and bioinformatics, Statistical methods

## Abstract

De novo transcriptome assembly from billions of RNA-seq reads is very challenging due to alternative splicing and various levels of expression, which often leads to incorrect, mis-assembled transcripts. BayesDenovo addresses this problem by using both a read-guided strategy to accurately reconstruct splicing graphs from the RNA-seq data and a Bayesian strategy to estimate, from these graphs, the probability of transcript expression without penalizing poorly expressed transcripts. Simulation and cell line benchmark studies demonstrate that BayesDenovo is very effective in reducing false positives and achieves much higher accuracy than other assemblers, especially for alternatively spliced genes and for highly or poorly expressed transcripts. Moreover, BayesDenovo is more robust on multiple replicates by assembling a larger portion of common transcripts. When applied to breast cancer data, BayesDenovo identifies phenotype-specific transcripts associated with breast cancer recurrence.

## Introduction

With the rapid development of massively parallel cDNA sequencing technologies, RNA sequencing (RNA-seq) has become an important technique for cancer-associated transcriptome analysis^1–4^. RNA-seq makes it possible to explore the complex transcriptomic landscapes at the resolution of single nucleotides, even in the absent of reliable reference genomes or transcriptomes. Thus, it allows the detection of known and novel transcripts with high sensitivity and accuracy.

However, transcriptome assembly from billions of short reads generated by RNA-seq is nontrivial. The main challenges are due to alternative splicing and variable levels of expression. In particular, alternative splicing, in which multiple transcripts are encoded via different combinations of exons from a single gene, often makes it impossible to directly link exons to transcripts. This problem is exacerbated by variable expression levels of alternatively spliced transcripts, where poorly-expressed transcripts are likely to be missed and highly-expressed transcripts suffer from high multiplicity due to sequencing errors in the corresponding vast number of short reads.

Several transcriptome assembly methods have been proposed over the past few years. In general, they can be categorized as reference-based assemblers and de novo assemblers. Reference-based assemblers, such as Cufflinks^5^ and Bayesembler^6^, first align the sequencing reads to a reference genome using splice-aware aligners, such as TopHat2^7^, and then merging the overlapping reads for each locus to build a graph, the paths through which correspond to the predicted transcripts. Reference-based assemblers rely on high quality reference genomes, which are usually unavailable for cancer research due to cancer cell genome alterations. In the absence of a reliable reference genome, a de novo transcriptome assembler may be used.

Most de novo transcriptome assemblers build de Bruijn graphs from the RNA-seq reads, and then identify transcripts heuristically as paths within the graphs. Trinity^8^ is the first transcript assembly method to apply de Bruijn graphs in this way. It starts by identifying and extending short k-mers into long contigs, builds de Bruijn graphs from the contigs, and finally searches for paths in each graph. Trinity greatly improved transcript assembly compared to earlier de novo assemblers based on genome-assembly methods; however, it is highly susceptible both to false positives, due to the exhaustive enumeration strategy used in path identification, and to sequencing errors. To reduce such errors, Oases^9^ applies a set of static and dynamic filters to the de Bruijn graph, followed by an exhaustive enumerating method to reconstruct transcripts. SOAPdenovo-trans^10^ integrates the ideas of Trinity, Oases, and the genome assembly method SOAPdenovo2^11^ by building a de Bruijn graph using SOAPdenovo2, removing errors in the graph using Trinity, and assembling the transcripts using the Oases graph traversal method. Although superior to previous approaches, Oases and SOAPdenovo-trans still produce many false positives because of their exhaustive searching algorithms. IDBA-tran^12^ removes erroneous vertices and edges from the graphs probabilistically and iteratively identifies transcripts by varying the k-mer length to deal with uneven transcript expression levels. When identifying transcripts, IDBA-tran searches for at most three maximum coverage paths. However, the top 3 maximum coverage paths might not correspond to the true set of transcripts. Moreover, some genes may undergo multiple alternative splicing events, especially for cancer cells. Therefore, identifying no more than three candidates may miss some transcripts. Bridger^13^ addresses this problem by combining Trinity and Cufflinks, where Cufflinks is applied on the graph built by Trinity to find minimal sets of transcripts. Cufflinks is more effective than exhaustive enumeration at reducing the number of assembled transcripts, but may still miss true solutions that could better explain the coverage and the idea of minimum set of paths does not have biological support.

Here we describe BayesDenovo for more accurate de novo transcriptome assembly from RNA-seq data. Building upon existing methods, BayesDenovo applies a read-guided strategy to construct splicing graphs from de Bruijn graphs, which can greatly reduce the false paths and connections caused by short k-mers. After constructing the splicing graphs, we further employ a generative Bayesian model introduced in Bayesembler^6^ to assemble reliable transcripts. Unlike the deterministic approach used in Bridger, the Bayesian model explains the observed reads based on the existence of transcripts, which will not directly penalize the transcripts by relative expression. Therefore, the transcripts with lower expression can be identified in a probabilistic manner. Simulation studies demonstrate that BayesDenovo significantly improves transcriptome assembly, especially on genes with more isoforms or with very high or low levels of expression. Studies on RNA-seq data for three MCF7 cell lines likewise demonstrate that BayesDenovo outperforms existing assemblers. BayesDenovo reports fewer transcripts than other assemblers on all three samples, while the number of successfully reconstructed transcripts is comparable to that of the most sensitive methods. Moreover, BayesDenovo is more robust: the assembled transcripts from multiple replicates more consistent than for other methods. When applied to breast cancer RNA-seq data, BayesDenovo identifies phenotype-specific transcripts associated with breast cancer recurrence. The assembled phenotype-specific transcripts are enriched in cell cycle, DNA damage, cell adhesion, and signaling pathways, shedding light on underlying mechanisms driving breast cancer recurrence.

## Results

### Simulated data

We conduct a comprehensive simulation study to compare BayesDenovo’s performance to existing assemblers. We generate a dataset of 80 million 100-bp paired-end strand-specific reads based on RefSeq^14^ human transcripts provided by the UCSC Genome Browser^15^ using the Flux simulator^16^, which simulates sequencing reads by mimicking the components in real RNA-seq experiments. Transcript expression levels are assigned randomly, including the possibility that some transcripts were not expressed. For benchmarking, we define as ‘expressed’ reference transcripts with simulated FPKM (Fragments Per Kilobases Per Million Fragments) > 1.

Using the simulated data, we compare the performance of BayesDenovo to the de novo assemblers: Bridger^13^, Oases^9^, Trinity^8^, IDBA-tran^12^, and SOAPdenovo-trans^10^, all of which use a pre-defined k-mer length of $k$ = 25. Unlike other methods, IDBA-tran supports multiple k-mer lengths; hence, we set *k* = 25 for IDBA-tran(Single) and *k* = 25, 27, 29 and 31 for IDBA-tran. Default values are used for other parameters.

Assembled transcripts are compared to reference annotations using BLAT^17^ with performance evaluated based on precision, recall, and F-score. An assembled transcript is defined as correct if at least 90% of its sequence overlapped with at least 80% of an expressed known transcript (as similarly defined in^13^). We define precision and recall as the percentage of correct transcripts among the assembled and reference transcripts, respectively, and Fscore, which assesses overall performance, as:

1 $$Fscore=\frac{2*precision*recall}{precision+recall}$$

**Figure 1:**
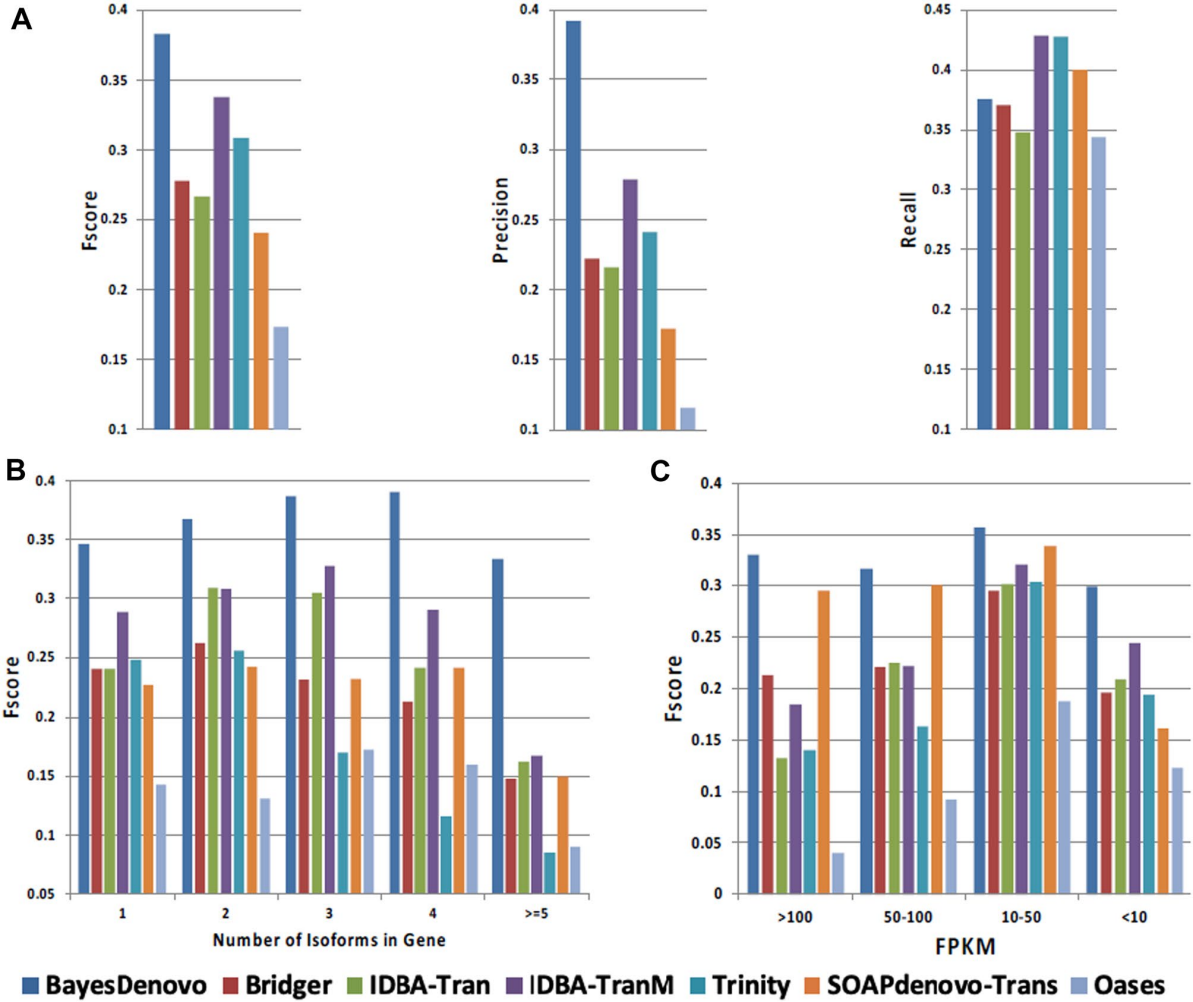
Performance comparison on transcript assembly using simulated data. **(A)** Overall performance evaluated by F-score, Precision and Recall. **(B)** Performance on subsets of genes grouped by the number of isoforms. **(C)** Performance on subsets of transcripts grouped by expression level.

As shown in Fig. 1A, BayesDenovo outperforms other methods as evaluated by Fscore, and exhibits much higher precision with recall comparable to other methods. In other words, BayesDenovo substantially reduces false positives with roughly similar numbers of correct transcripts. This is probably due to the read-guided strategy for splicing graph reconstruction, which deletes erroneous nodes and edges while retaining true transcripts. This improvement is critical for biological data, as it greatly reduces the number of candidate transcripts.

Figure 1B shows the performance based on alternative splicing and variable expression levels, which are major challenges in transcriptome assembly. Using BLAT, we group assembled gene transcripts according to their numbers of expressed transcripts, so that genes with 1, 2, 3, 4, and more than 4 expressed transcripts are evaluated separately. Reconstruction is generally more difficult for genes with more expressed transcripts, due to the increased complexity of the de Bruijn and splicing graphs. However, unlike other methods, BayesDenovo maintains robust performance across genes with different expression levels (Fig. 1B): On genes with ≥ 4 expressed transcripts, it exhibits a significant improvement (> 0.1 in Fscore) over other methods.

Figure 1C categorizes transcripts by expression level. For moderately expressed transcripts (10–50 FPKM), the performance of the 7 methods are comparable. However, for high-expressed transcripts and low-expressed transcripts, BayesDenovo achieves higher Fscores. Highly-expressed transcripts are associated with more complex de Bruijn and splicing graphs, for which BayesDenovo’s read-guided strategy may reduce false nodes and edges. For poorly-expressed transcripts are often missed by other assemblers during path searching, for which BayesDenovo’s Bayesian estimation strategy appears to be more effective.

Furthermore, we evaluate BayesDenovo with another two methods, rnaSPAdes^18^ and Trans-ABySS^19^, on another simulation dataset. We use the evaluation metrics published in rnaSPAdes^18^ to comprehensively compare all methods. The results show a similar finding that BayesDenovo assembles full-length transcripts more accurately than existing methods (see Supplemental Section S1.3 for details). We also test the computational time for the single k-mer assemblers. The speed of BayesDenovo is comparable to most assemblers (see Supplemental Section S1.4).

### Cell line data

We apply BayesDenovo and competing methods to three real RNA-seq samples, namely 76-bp paired-end RNA-seq data from three biological replicates of the MCF7 breast cancer cell line (GSM958745: SRR521521, SRR521522, SRR521523)^20^. For benchmarking, we combine the MCF-7 human breast cancer transcriptome detected by Pacific Biosciences^21^ with the RefSeq human transcripts as provided by the UCSC Genome Browser. As for simulated data, we use BLAT to compare the assembled transcripts with the combined transcriptome, and defined as matches assembled transcripts with ≥ 90% of sequence overlap with ≥ 80% of a known transcript. The *k*-mer length is fixed to 25 for all assemblers, except for IDBA-Tran for which the *k*-mer length ranged from 25 to 37 with step size 4. Default values are used for other parameters.

Because, for real data, it is impossible to know with certainty which transcripts are present, Table 1 reports the number of matched transcripts against the number of assembled transcripts for each method in each experiment. In general, the performance of most competing assemblers is consistent both across the three samples and with the simulation studies. On all three samples, BayesDenovo reports fewer transcripts than other assemblers, while successfully reconstructing a number of matched transcripts comparable to that of the most sensitive methods. Trinity reconstructs a few more matched transcripts by enumerating all of the paths in the graph, yet it reports many more transcripts, which dramatically decreases its accuracy. IDBA-tran is a bit more sensitive by virtue of updating the graph with alternative *k*-mer lengths; however, it still reports a huge number of transcripts (more than twice that of BayesDenovo). BayesDenovo exhibits much higher precision than do the other de novo assemblers.

**Table 1:** Performances of the assemblers on MCF-7 breast cancer cell line RNA-seq data.

		BayesDenovo	Bridger	IDBA-tran (single)	IDBA-tran	Trinity	SOAPdenovo-trans
Sample 1	No. of matched transcripts	9226	8148	6315	9638	9622	5159
	No. of assembled transcripts	21,922	81,201	56,212	54,623	101,366	237,815
	**Precision**	**0.421**	**0.1**	**0.112**	**0.176**	**0.095**	**0.022**
Sample 2	No. of matched transcripts	9348	8011	6021	9503	9348	7469
	No. of assembled transcripts	22,998	77,220	54,722	57,107	96,500	110,179
	**Precision**	**0.406**	**0.104**	**0.11**	**0.166**	**0.097**	**0.068**
Sample 3	No. of matched transcripts	9012	8200	7718	10,103	9027	7522
	No. of assembled transcripts	20,171	53,970	43,793	42,151	68,816	70,859
	**Precision**	**0.447**	**0.152**	**0.176**	**0.24**	**0.131**	**0.106**

Bold numbers show the metric where BayesDenovo significantly outperforms existing methods. Bold underline numbers highlight the performance of BayesDenovo.

We leverage the large degree of overlap expected among the transcriptomes of biological replicates to evaluate the de novo assemblers. To compare the assembly results from multiple samples to the reference simultaneously, we first use GMAP^22^ to align (in GTF format) the assembled transcripts to the GRCh37/hg19 human reference genome^15^. We then use Cuffcompare from the Cufflinks package^5^ to label the assembled transcripts from the three samples with the combined transcriptome as the reference. We consider a transcript as correct when its full intron chain was identified (i.e., all intron boundaries matched exactly). In addition to the de novo assemblers, we also apply the reference-based assembler Cufflinks to the three samples, where the sequencing reads are first mapped to the GRCh37/hg19 human reference genome using Tophat2^7^. To assess consistency across replicates, we merge the transcripts reconstructed in the three samples by each assembler, and examine transcripts common to all three replicates. In general, deterministic approaches will be more sensitive to noise or variation existed in individual samples. Therefore, we expect that the existing methods will be less stable when analyzing multiple samples. Unlike deterministic approaches, BayesDenovo utilizes a Bayesian model to capture the observed reads and identify assembled transcripts by a sampling approach, which is expected to be more robust to individual noise. Table 2 lists the number of common transcripts and the total number of transcripts among the three replicates. BayesDenovo exhibits the highest consistency among assemblers by detecting fewer transcripts relative to the number of common ones. In this regard, BayesDenovo is even better than the reference-based Cufflinks method. Therefore, BayesDenovo is both more accurate and more robust in dealing with multiple replicates.

**Table 2:** Comparison of consistency across three biological replicates.

	BayesDenovo	Bridger	IDBA-tran (single)	IDBA-tran	Trinity	SOAPdenovo-trans	Cufflinks
No. of common transcripts	6098	6771	5130	7053	8364	6248	7497
Total number of transcripts	45,184	173,377	116,951	118,860	216,695	337,712	65,393
**Proportion of common transcripts**	**0.135**	**0.039**	**0.044**	**0.059**	**0.039**	**0.019**	**0.115**

Bold numbers show the metric where BayesDenovo significantly outperforms existing methods. Bold underline numbers highlight the performance of BayesDenovo.

**Figure 2:**
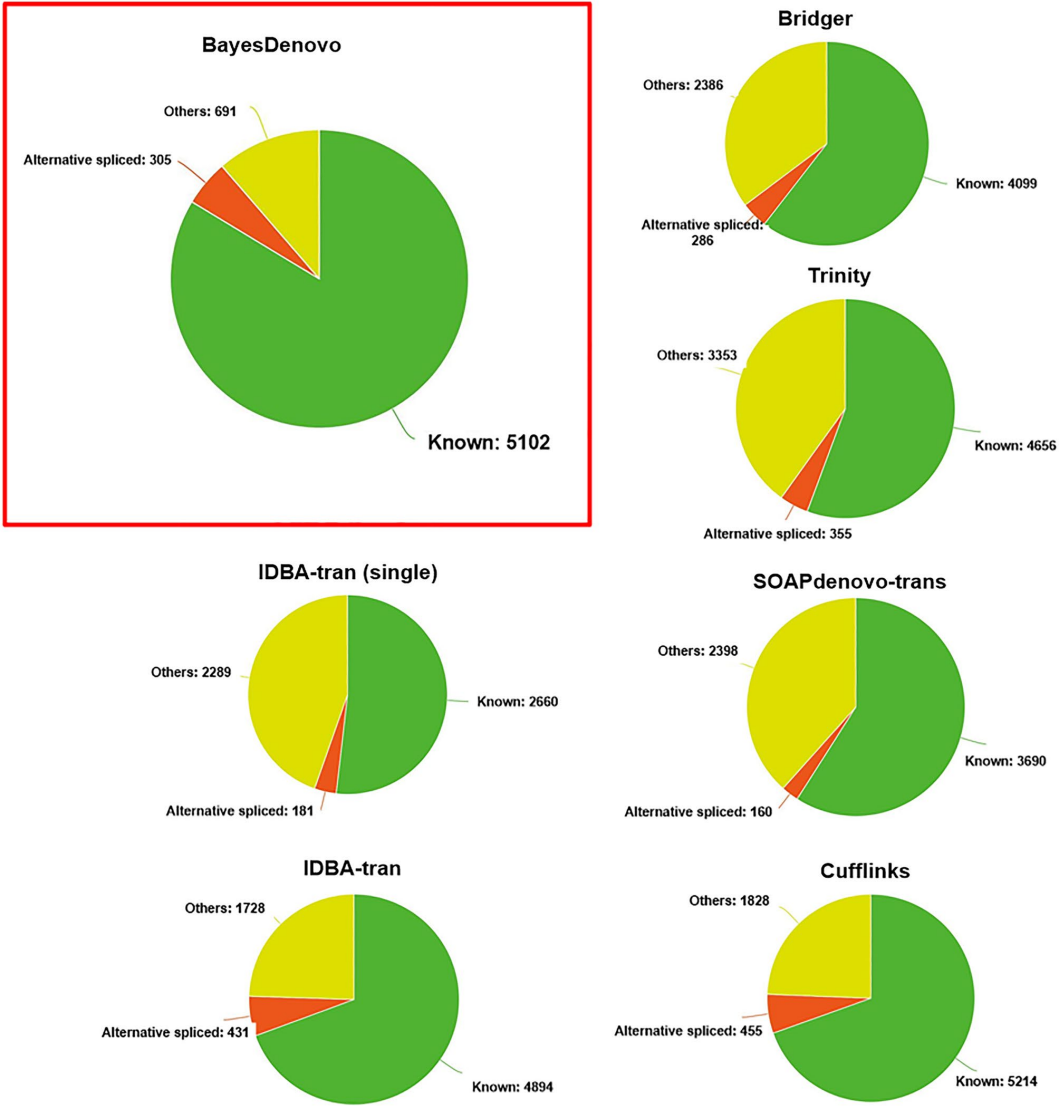
Transcripts assembled by competing methods that are common to the three cell line replicates in the study.

Though the transcripts present in the MCF-7 cells are unknown, it is reasonable to assume that transcripts identified in all three replicates are more likely correct. Figure 2 categorizes the common transcripts according to their structure compared to the reference annotation. We also report potentially novel isoforms where at least one splice junction is shared with a reference transcript, which may correspond to unknown alternatively spliced transcripts. BayesDenovo reconstructs more correct transcripts (*N* = 5102) than the other de novo assemblers. Though Cufflinks identifies a few more (*N* = 5214), it assembles more transcripts in the ‘Others’ category, which are less likely true in terms of structure. Hence, BayesDenovo is more robust and better detects true transcripts than other assemblers.

### Breast cancer recurrence study

We apply BayesDenovo to breast cancer data acquired by The Cancer Genome Atlas (TCGA) project^23^. The study is designed to identify phenotype-specific transcripts associated with breast cancer recurrence by reconstructing the transcripts in the cancer samples without a reference genome. 18 estrogen receptor positive (ER +) tumors from patients are collected for this study, where 8 patients are dead within 5 years, labeled as ‘early recurrence’; 10 patients are still alive with follow up longer than 5 years, labeled as ‘late/non recurrence’.

**Figure 3:**
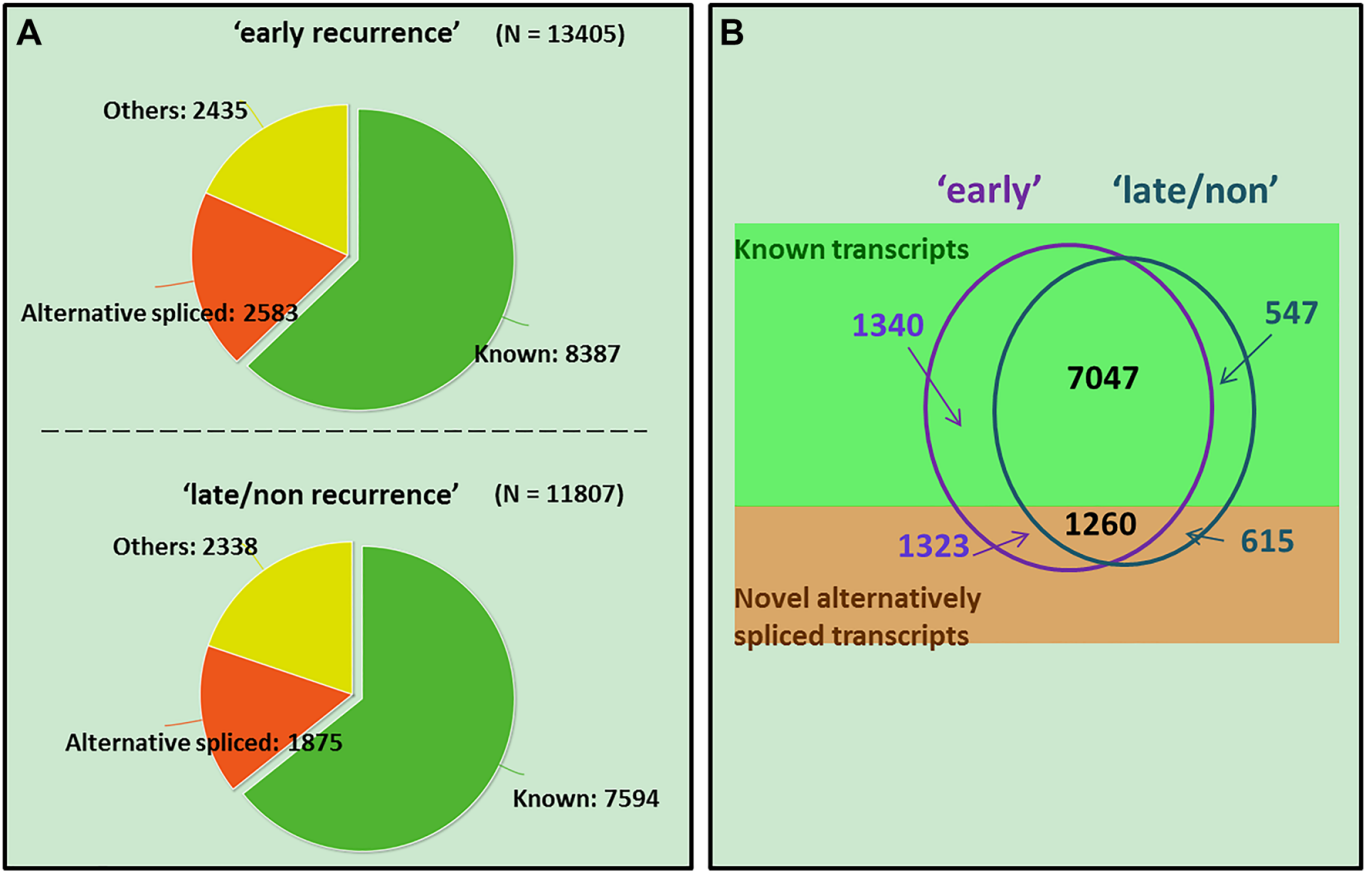
Assembled transcripts in ‘early-recurrence’ breast cancer samples and ‘late/non-recurrence’ samples.

We download the sequencing data (Level 1) profiled by Illumina HiSeq 2000 RNA Sequencing Version 2 from the TCGA data portal and use BayesDenovo to assemble the transcripts for each tumor sample. Then, by aligning the assembled transcripts to the reference genome (hg19) using GMAP, we compare the assembled transcripts from multiple samples using Cuffcompare. For each phenotype, a transcript is detected as expressed if it is assembled in at least half of the samples (i.e., 4 or more samples in the ‘early recurrence group’ and 5 or more samples in the ‘late/non recurrence’ group). This detects 13,405 transcripts in the ‘early recurrence’ group, and 11,807 in the ‘late/non recurrence’ group. Figure 3A compares the reconstructed transcripts with the known RefSeq human transcripts, revealing that 62.6% of transcripts are in the ‘early recurrence’ group, 64.3% in the ‘late/non recurrence’ group, and around 20% in each group are novel alternatively spliced transcripts (Fig. 3A). Figure 3B further compares the known and novel alternative spliced transcripts between the two phenotypes. Most of the transcripts are common to both groups, while 2663 transcripts in the ‘early recurrence’ group and 1162 in the ‘late/non-recurrence’ group are phenotype-specific, including 1323 and 615 novel alternatively spliced transcripts, respectively.

**Figure 4:**
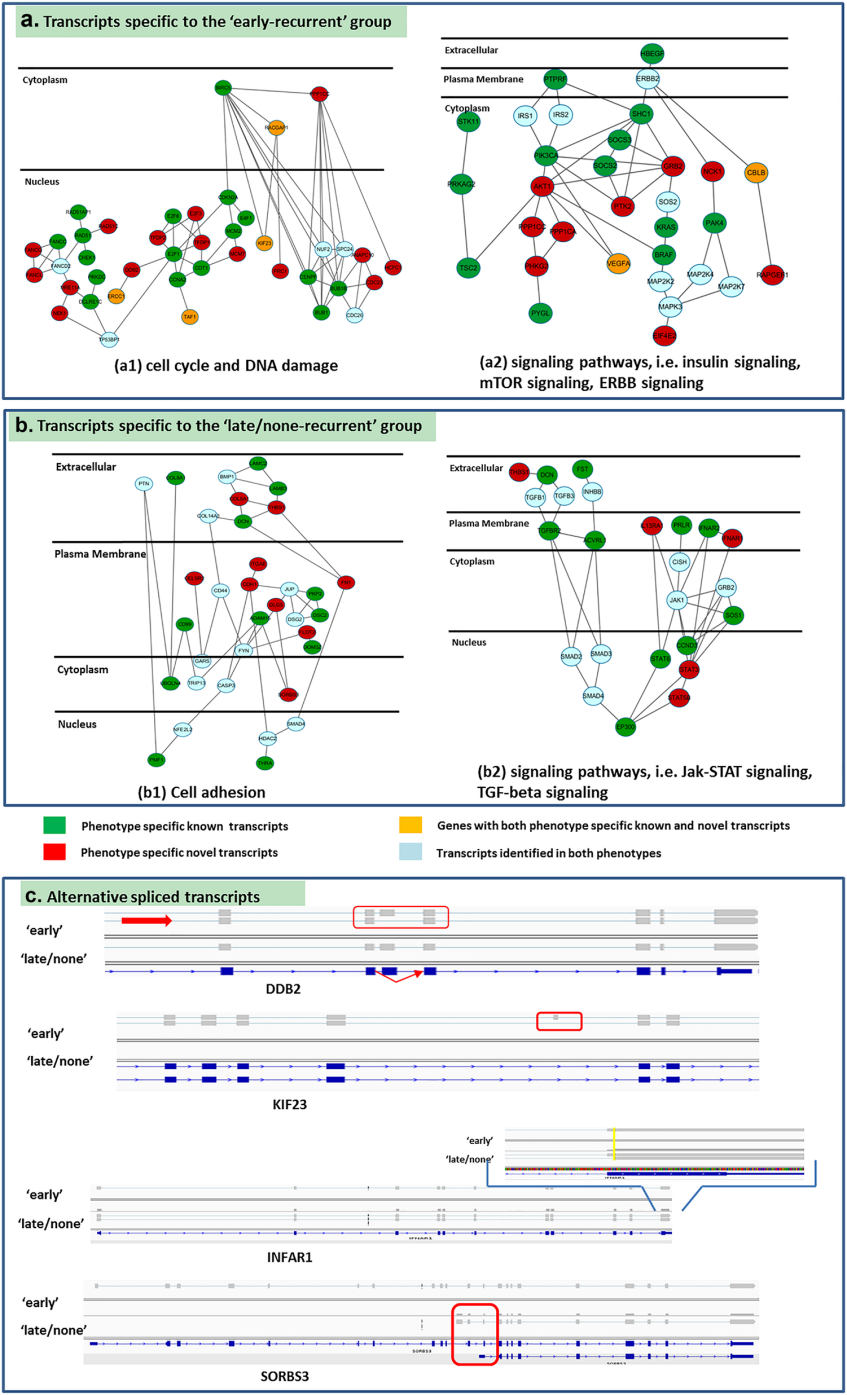
Phenotype-specific transcripts assembled by BayesDenovo: **(a)** transcripts specific to the ‘early-recurrence’ group enriched in cell cycle, DNA damage and signaling pathways; **(b)** transcripts specific to the ‘late/none-recurrence’ group enriched in cell adhesion and signaling pathways. **(c)** Examples of novel alternative spliced transcripts.

Functional analysis of the phenotype-specific transcripts reveals major cellular functions associated with breast cancer. Transcripts specific to the ‘early-recurrence’ samples are enriched in cell cycle, DNA damage, and signaling pathways such as Insulin signaling, mTOR signaling, and ERBB signaling (Fig. 4a); transcripts specific to the ‘late-recurrent’ samples are enriched in cell adhesion and signaling pathways such as Jak-STAT signaling and TGF $\upbeta$ signaling (Fig. 4b). In the above-mentioned cellular functions, several phenotype specific transcripts (as shown in Fig. 4c) are novel (i.e., alternative spliced transcripts absent from the known transcriptome). For example, a novel transcript of DDB2 due to exon skipping is detected specific to the ‘early-recurrent’ group. DDB2 is associated with breast tumor invasion and is a novel regulator of NF-$\upkappa$ B, thereby affecting expression of its target genes^24^. KIF23 has both a known transcript and a novel transcript due to intron retention as assembled in the ‘early-recurrence’ group. Overexpression of KIF23 is correlated with poor survival of patients with ER-positive breast cancer^25^. A novel IFNAR1 transcript detected in the ‘late/none-recurrent’ group has an alternative 3’ accept site; the canonical IFN $\mathrm{\alpha }$ signaling pathway is involved in metastasis^26^ and aromatase inhibitor resistance in breast cancer^27^. A novel transcript of SORBS3 is more complex in terms of alternative splicing; SORBS3 is also associated with poor outcomes of patients and its product has tumor suppressive activities^28^.

## Conclusions

BayesDenovo is a de novo assembler that accurately reconstructs transcripts from short RNA-seq reads. It is designed to tackle the problem of high false positives, which is a serious problem with conventional transcriptome assemblers. Using a read-guided strategy, BayesDenovo can construct splicing graphs of higher quality by cutting down on false nodes and edges while retaining information regarding true transcripts. The read-guided strategy provides an important advantage, especially for high-expressed transcripts which are more likely to be affected by sequencing errors. Using a Bayesian approach to estimate transcripts from the splicing graphs, BayesDenovo can detect a set of transcripts that better explain reads in the graph and thus is more effective on genes with alternative spliced transcripts or with poorly-expressed transcripts.

When applied both to simulated data and to real data with benchmarks, BayesDenovo consistently outperforms existing assemblers. Simulation studies demonstrate that BayesDenovo can significantly reduce false positives and improve overall performance, as measured by F-score. Specifically, the precision of BayesDenovo is much higher than other methods, while the recall is comparable. Moreover, BayesDenovo provides a significant advantage for genes with highly- or poorly-expressed or alternative transcripts. A cell line study has further demonstrated the superiority of BayesDenovo to other assemblers on real RNA-seq data. In all three replicates, BayesDenovo reports much fewer candidate transcripts with comparable numbers of true ones—even for the most sensitive method Trinity. The robustness of BayesDenovo was demonstrated by a larger overlap of assembled transcripts among three replicates.

We have applied BayesDenovo to breast cancer RNA-seq data to identify phenotype-specific transcripts. Functional analysis of transcripts in the ‘early-recurrence’ and ‘late-recurrence’ groups points to major cellular functional differences associated with breast cancer, which may shed light on molecular mechanisms underlying breast cancer recurrence.

Due to difficulties in sequence similarity and expression variation, it is very challenging to accurately assemble transcripts from short read RNA-seq data. The development of new long read sequencing platform such as Pacific Biosciences (https://www.sciencedirect.com/science/article/pii/S1672022915001345) and Nanopore technology (https://www.nature.com/articles/s41592-019-0617-2), makes it possible to sequence the whole transcripts in one read, which can significantly improve the accuracy of transcript identification. However, the throughput of long read sequencing platform is usually low and false negative rate will be a major issue. Therefore, a hybrid transcriptome assembly integrating both long and short sequences will be the future direction for de novo assemblers. Our BayesDenovo framework can be further improved to incorporate long reads. For example, the long reads can be very helpful for the step of splicing graph construction to build the major contigs. The short reads can then be further used to find branches extension from the major contigs.

## Methods

### BayesDenovo overview

**Figure 5:**
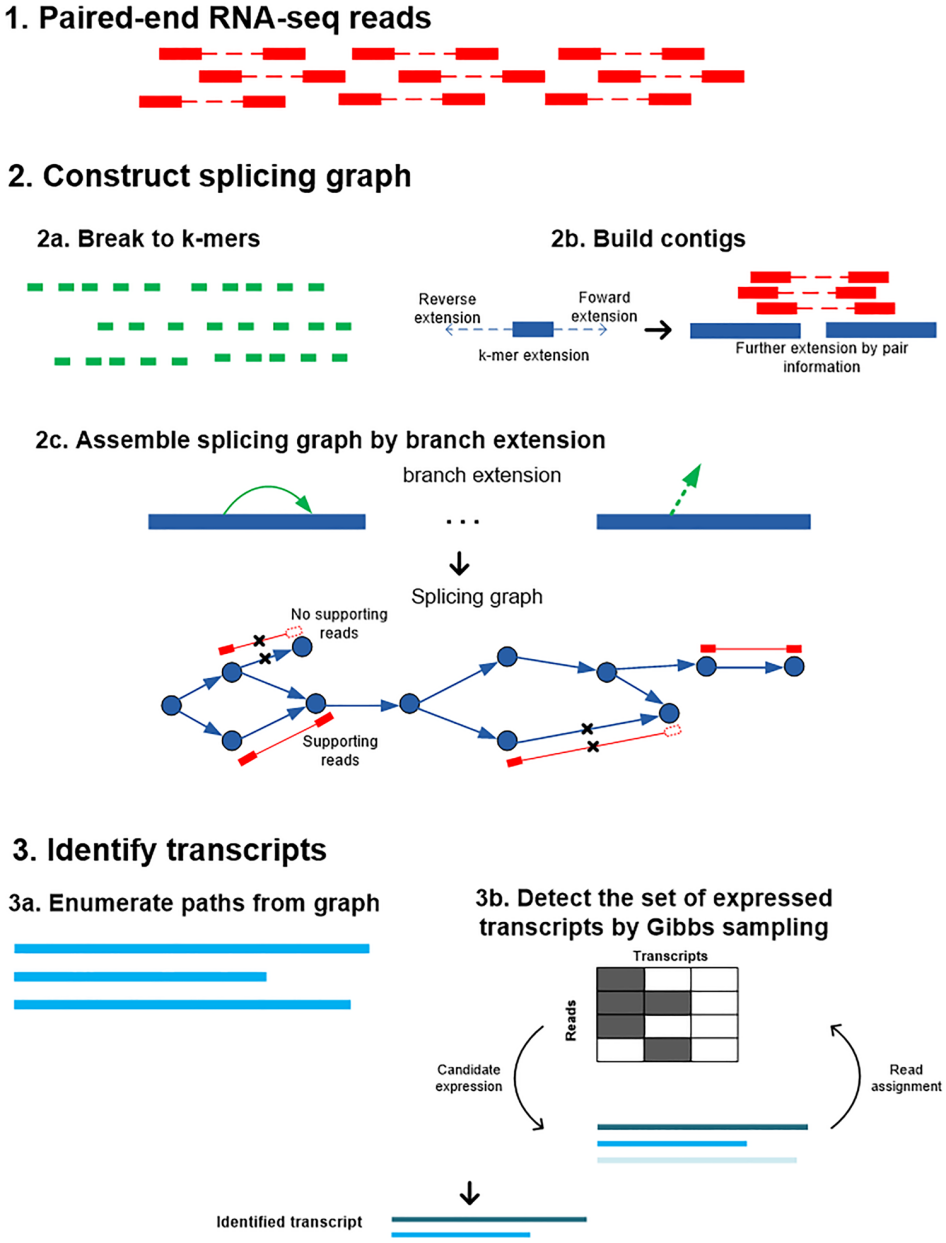
An Overview of the BayesDenovo approach. The approach consists of the following two major steps: (1) splicing graph construction from short RNA-seq reads; (2) transcript estimation from the splicing graphs.

BayesDenovo aims to accurately assemble transcripts directly from RNA-seq reads. Incorporating ideas from existing methods, BayesDenovo consists of two steps: (1) splicing graph construction from short RNA-seq reads; and (2) transcript estimation from the splicing graphs. An overview of the BayesDenovo approach is shown in Fig. 5 and the approach consists of: (1) constructing splicing graphs from RNA-seq reads; and (2) detecting transcripts from the graphs. Splicing graphs are constructed by breaking sequencing reads into k-mers, extending the k-mers into contigs, and then further extending the branches of the contigs in a read-guided way, so as to retain only those branches and connections supported by sequencing reads. In this way false nodes and edges in the splicing graph are greatly reduced, especially for highly expressed, alternatively spliced genes. When assembling transcripts from splicing graphs, BayesDenovo models variable expression levels using the approach implemented in Bayesembler^6^. For each splicing graph, a set of candidate transcripts are enumerated and the true transcripts are estimated in a Bayesian framework. Specifically, a sampling procedure is designed to iteratively estimate the set of expressed transcripts, their abundance, and the probability of each read being assigned to each transcript. This greatly increases the detection of poorly-expressed transcripts, which other path traversing methods tend to penalize.

### Construction of splicing graph

Genes with alternative spliced transcripts can be represented by splicing graphs, in which nodes correspond to exons (i.e., bunches of common exons) and edges represent splicing junctions. BayesDenovo uses a read-guided strategy to construct splicing graphs from *k*-mers generated from RNA-seq reads, as follows.

First, as in Trinity and Bridger the sequencing reads are broken into *k*-mers, which are saved in a hash table. Erroneous *k*-mers are removed by the same strategy as in Trinity. Second, a most frequent *k*-mer, with Shannon’s entropy $H>1.5$ and frequency > 1, is selected as a seed, which is then extended repeatedly in two directions by finding the most frequent unused *k*-mer that has a *k*-1 base suffix that overlaps with the *k*-1 bases prefix of the current contig. Third, when the contig can no longer be extended by overlapping *k*-mers, paired-end information of the sequencing reads is used to further extend the contig. Fourth, after building the main contig, branch extension is performed, in a read-guided way, on *k*-mers for contigs that have alternative extensions. Specifically, for branch extension, an overlapping *k*-mer is first obtained from the reads overlapping with the current contig. If no overlapping *k*-mers are found from overlapping reads, an overlapping *k*-mer at the end of non-overlapping reads is used to extend the contig. Alternative paths are added in by using overlapping *k*-mers and paired-end information until the path can no longer be extended or until reaching a previously used *k*-mer in the current graph. By virtue of the read-guided strategy, extended branches are supported by sequencing reads, while the false connections, which lack supporting reads, will not be added as alternative paths.

As a result, a set of splicing graphs of high accuracy are constructed directly from the sequencing reads, taking advantage of the branch extension strategy that incorporates read information. Without loss of generality, each splicing graph corresponds to the genomic locus of a gene. Graphs for highly expressed genes are more likely to suffer from erroneous nodes and edges, where the read-guided strategy will greatly improve the quality of splicing graphs.

### Transcripts estimation

Transcripts are reconstructed from splicing graphs probabilistically by modeling the abundance of potential transcripts within a Bayesian framework. For this, BayesDenovo employs the model implemented in Bayesembler, an ab initio transcriptome assembly method relying on a reference genome. Expressed transcripts are estimated from each splicing graph, as follows.

First, a set of candidate transcripts are constructed by iteratively traversing paths and pruning those edges with lowest coverage until the total number of candidates is ≤ 100. Second, using a hidden binary random variable to model whether a candidate transcript is expressed or not, the Bayesian framework jointly models the set of expressed transcripts, the abundance of expressed transcripts, and the probability for read assignment to the transcripts. A Gibbs sampling procedure estimates the frequency at which transcripts are observed from their posterior distributions, and determines the set of transcripts that best explain the observed sequencing reads associated with the graph. By modeling the existence of the candidate transcripts with a hidden variable, this Bayesian framework greatly increases efficiency in detecting poorly-expressed transcripts, which are likely to be penalized by other path traversing methods. The details of the Bayesembler framework is described in Supplemental Sects. 1.1 and 1.2.

## Supplementary Information

Supplementary Information.

## Data Availability

The C++ source code of BayesDenovo is available at https://github.com/henryxushi/BayesDenovo. Contact: xuan@vt.edu.
