# Supplementary material for: A Bayesian approach for accurate de novo transcriptome assembly
Source: Sci Rep. 2021 Sep 3;11:17663. doi: 10.1038/s41598-021-97015-x (PMC8417280; doi:10.1038/s41598-021-97015-x)
Supplement: Supplementary file 1 — Supplementary Information. [file 41598_2021_97015_MOESM1_ESM.pdf]

Supplemental Material

A Bayesian approach for accurate *de novo* transcriptome assembly

Xu Shi, Xiao Wang, Andrew F. Neuwald, Leena Halakivi-Clarke, Robert Clarke, and Jianhua Xuan

Supplement to Methods

S1.1 Splicing graph construction

In BayesDenovo, we develop the splicing graph construction based on the strategy used in Trinity [1] and Bridger [2]. First, we process k-mers from all the reads to build a hashtable indexed by k-mers. Erroneous k-mers will be removed by low frequency (frequency < 2) and Shannon's entropy (H<1.5). This choice of parameters is validated in [2]. For the k-mer extension, we will start from the most frequent k-mer and extend one base at a time by finding another k-mer with consecutive k-1 matches. We will continue this extension at both directions until no extension can be further applied. After assembling the contigs, we will check if any paired-end reads are mapped to different contigs. If there are multiple reads supporting the connection of two contigs, we will combine the two contigs into one contig as shown in Figure S1. Since we employ the bayesembler model [3] in the transcript estimation step, the paired end reads will be further evaluated by mapping compatibility.

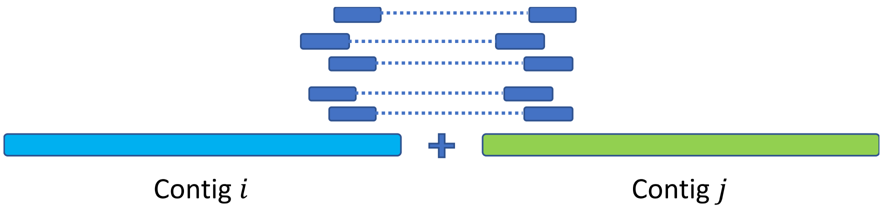

Figure S1. Example of contig concatenation by paired-end reads support

For alternative extensions occurred during the contig extension, we will remove erroneous extensions by a read guided strategy. Due to the complexity of the k-mers, some alternative extensions may be only supported by part of reads or mixed sequences from multiple reads. Therefore, we only keep the extensions that are supported by full-length read, which will effectively filter out false positive paths in the graph. Figure S2 shows an example of how the read guided strategy works for removing erroneous branches. This example shows a 5-mer de Brujin graph where the red branch is constructed by the last three reads that happen to have the 5-mers to extend. In our graph construction step, the red branch will be removed since it is not supported by a full length read.

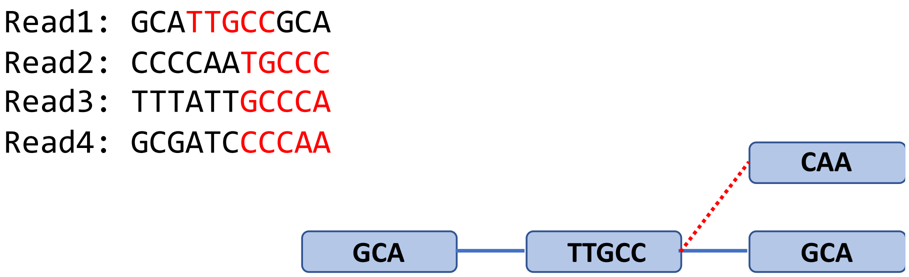

Figure S2. Example of read-guide branch correction strategy

S1.2 Bayesian framework to identify transcript structure from splicing graph

In BayesDenovo, we employ a Bayesian framework published in [3] to reliably identify transcript structures from splicing graph. The framework utilizes a Bayesian model to simulate the generation of sequencing reads. First, a candidate set of isoforms will be enumerated from the

splicing graph. The existence of each isoform will be modeled as a binary variable z. If one isoform is actually expressed in the data, the associated variable z for this isoform will be 1. Otherwise, z will be 0 representing the isoform is enumerated by mistake. Due to the complexity of splicing graph, the size of candidate isoform set will be huge. Therefore, we can expect the vector $\boldsymbol{z}$ of all candidate isoforms will be sparse. The sparsity will be modeled by a Bernoulli distribution with a small probability of success.

$$p(\boldsymbol{z}) \sim \prod_{t} p(z_t;\theta) \sim \prod_{t} \theta^{z_t}(1-\theta)^{1-z_t},$$

where $\theta \ll 1$ introduces the sparsity in variable $\boldsymbol{z}$. Suppose the total expression of isoforms equals to 1, then we can model the relative expression of isoform by Dirichlet distribution given the existence status of isoforms:

$$p(\boldsymbol{x}|\boldsymbol{z},\gamma) = \frac{\Gamma(b_z\gamma)}{\Gamma(\gamma)^{b_z}} \prod_{t=1}^{b_z} x_t^{\gamma-1},$$

where $\boldsymbol{x}$ is the relatively expression for all isoforms, b_z is the total number of expressed isoforms and γ is a hyper parameter. For a given transcript, the probability of a paired reads that can be generated from this transcript can be modeled by a generative model with three parts. Suppose we would like to generate a fragment or paired-end reads $r = (r_1, r_2)$ from transcript t. We need to first determine the three prime position s of the fragment, which can be sampled from

$$p(s|t,\mu,\sigma) \sim \frac{1}{K} \sum_{i=1}^{s} N(i;\mu,\sigma) \quad \text{s.t. } s \le l_t$$

where l_t is the transcript length, K is a normalization factor and $N(x;\mu,\sigma)$ represents the fragment length distribution with mean μ and standard deviation σ estimated from genes with single-isoform structure. Then the length of the fragment can be sampled from

$$p(l_r|s) \sim N(l_r;\mu,\sigma) \quad \text{s.t. } l_r \le s$$

Finally, the sequence quality is incorporated into the model by measuring the quality of each base of r_1 and r_2.

The full joint model of the Bayesian framework will be estimated by a Gibbs sampling approach. The two key parameters $\boldsymbol{z}$ and $\boldsymbol{x}$ will be iteratively sampled. The confidence of existence for an isoform will be estimated as the frequency of z_t equals to 1. The isoforms with confidence of existence larger than 0.5 will be classified as existed isoforms in the final identified isoform sets by default.

S1.3 Assemblers evaluation by rnaQUAST

Beside the metrics used in Figure 1 in the main text, we further comprehensively evaluate the assemblers using another evaluation package called rnaQUAST [4] on another simulation data generated by the Flux simulator. Additionally, we add rnaSPAdes [5] and Trans-ABySS [6] in our comparison study. The results are shown in Table S1.

Supplemental Table S1. Performance evaluated by rnaQUAST

**Assemblers**	**BayesDenovo**	**Bridger**	**rnaSPAdes**	**Trinity**	**IDBA_Tran**	**IDBA_TranM**	**Oases**	**Trans-ABySS**
Transcripts	15889	18293	21679	24113	20604	19728	37751	28227
Transcripts > 1000 bp	11042	10637	10787	9868	10702	11111	19589	11592
Avg. alignment length	1960.14	1728.27	1564.574	1315.244	1747.193	1882	1623.185	1290.218
Misassemblies	280	777	362	239	237	232	1342	129
50%-assembled isoforms	9433	9020	9679	8407	9289	9414	10179	10574
95%-assembled isoforms	5722	5422	5884	2532	5280	5838	4685	5718

Based on average alignment length, we can see that our BayesDenovo method assembles longer transcripts than existing methods. Similar to the results shown in Figure 2 in the main text, Bayesdenovo detects full length transcripts (95%-assembled isoforms) more accurately. It has assembled 5722 full length transcripts isoforms from only 15889 total predictions.

S1.4 Computational time evaluation

The computation time of single k-mer assemblers is evaluated on a high-performance cluster. For assemblers with multiple threads support, we use 10 threads for this evaluation. Figure S3 shows the computational time on a simulation dataset with 80 million reads. It can be seen that BayesDenovo is comparable to other assemblers even using a sampling based approach.

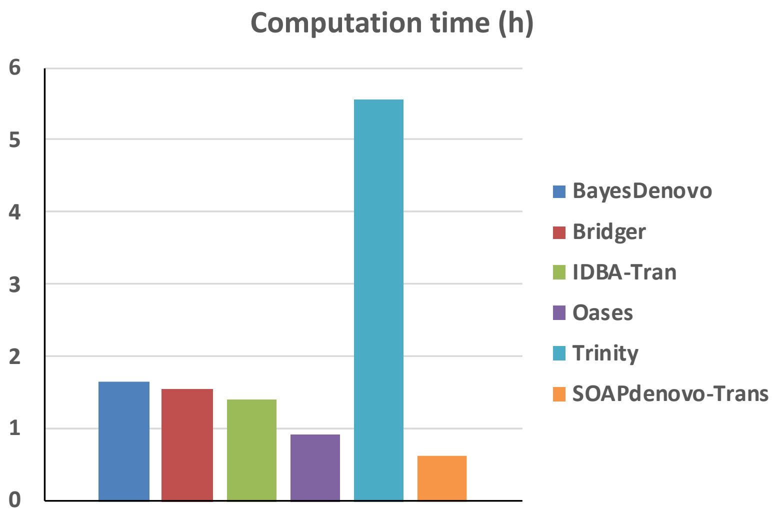

Figure S3. Computational time evaluation

References

[1] Grabherr, Manfred G., et al. "Full-length transcriptome assembly from RNA-Seq data without a reference genome." *Nature biotechnology* 29.7 (2011): 644.

[2] Chang, Zheng, et al. "Bridger: a new framework for de novo transcriptome assembly using RNA-seq data." *Genome biology* 16.1 (2015): 1-10.

[3] Maretty, Lasse, Jonas Andreas Sibbesen, and Anders Krogh. "Bayesian transcriptome assembly." *Genome biology* 15.10 (2014): 1-11.

[4] Bushmanova, Elena, et al. "rnaQUAST: a quality assessment tool for de novo transcriptome assemblies." *Bioinformatics* 32.14 (2016): 2210-2212.

[5] Bushmanova, Elena, et al. "rnaSPAdes: a de novo transcriptome assembler and its application to RNA-Seq data." *GigaScience* 8.9 (2019): giz100.

[6] Robertson, Gordon, et al. "De novo assembly and analysis of RNA-seq data." *Nature methods* 7.11 (2010): 909-912.
